# Supplementary material for: Identification of photoperiod-induced specific miRNAs in the adrenal glands of Sunite sheep (Ovis aries)
Source: Front Vet Sci. 2022 Jul 22;9:888207. doi: 10.3389/fvets.2022.888207 (PMC9354845; doi:10.3389/fvets.2022.888207)
Supplement: Supplementary file 1 [file Data_Sheet_1.ZIP › Supplementary file/miRDeep2 file.zip!/miRDeep2 file/Know_Structure/SP42A3/oar-mir-329b.pdf]

miRBase precursor : oar-mir-329b
Total read count : 1060
oar-miR-329b-5p read count : 21
oar-miR-329b-3p read count : 1035
remaining reads : 4

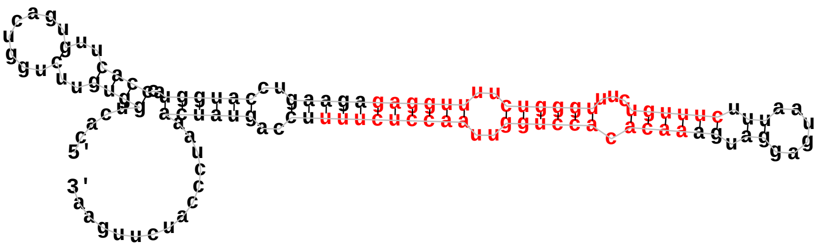

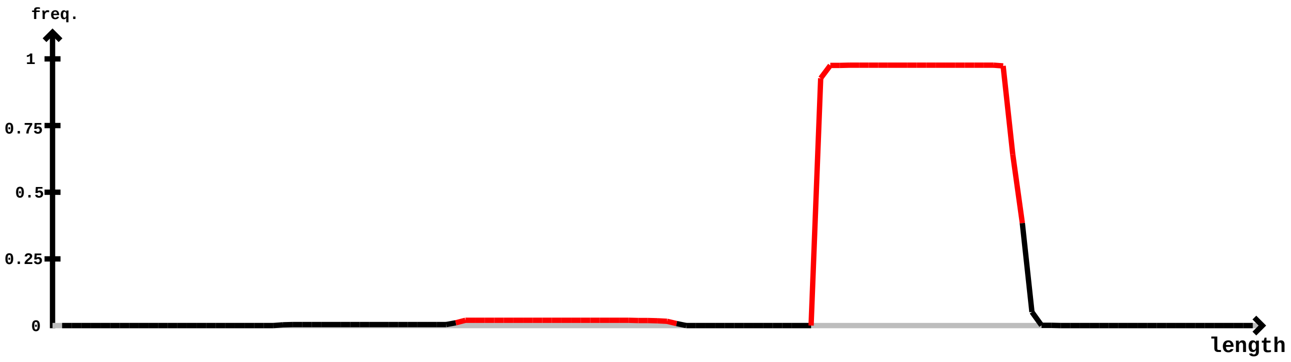


```
                                                                              oar-miR-329b-3p
                                         oar-miR-329b-5p
5'- cacugguguucuggucaguguucacccaugguaccugaagagagguuuucuggguuucuguuucuuuaaugaggaugaaacacaccugguuaaccucuuuuccaguaucaaaucccaucuugaa   -3'    exp
    ....((((..(........)..))))..((((((..(((((((((((..((((((...((((((.((......)).)))))).))))))..)))))))))))..))))))..............    reads      mm
    ......................cacccaugguaccugaaga...................................................................................    3          0
    .......................acccaugguaccugaaga...................................................................................    1          0
    ........................................agagguuuucuggguuucu.................................................................    1          0
    ........................................agagguuuucuggguuucugu...............................................................    1          0
    ........................................agagguuuucuggguuucuguu..............................................................    1          0
    ........................................agagguuuucuggguuucuguuu.............................................................    3          0
    ........................................agagguuuucuggguuucuguuuc............................................................    1          0
    .........................................gagguuuucuggguuucuguu..............................................................    1          0
    .........................................gagguuuucuggguuucuguuu.............................................................    6          0
    .........................................gagguuuucuggguuucuguuuc............................................................    7          0
    ..............................................................................aacacaccugguuaaccuc...........................    3          0
    ..............................................................................aacacaccugguuaaccucu..........................    349        0
    ..............................................................................aacacaccugguuaaccucuu.........................    273        0
    ..............................................................................aacacaccugguuaaccucuuu........................    339        0
    ..............................................................................aacacaccugguuaaccucuuuu.......................    19         0
    ...............................................................................acacaccugguuaaccucu..........................    2          0
    ...............................................................................acacaccugguuaaccucuu.........................    1          0
    ...............................................................................acacaccugguuaaccucuuu........................    14         0
    ...............................................................................acacaccugguuaaccucuuuu.......................    34         0
    .................................................................................acaccugguuaaccucuuuucc....................    1          0
```
